# Green Chemistry Teacher Professional Development in New York State High Schools: A Model for Advancing Green Chemistry

**DOI:** 10.1021/acs.jchemed.2c01173

**Published:** 2023-05-03

**Authors:** Amy S. Cannon, Kate R. Anderson, Mollie C. Enright, Donia G. Kleinsasser, Ann R. Klotz, Natalie J. O’Neil, Lucas J. Tucker

**Affiliations:** † Beyond Benign, 18 Church Street PO Box 1016, Wilmington, Massachusetts 01887, United States; ‡ Department of Chemistry and Biochemistry, School of Green Chemistry and Engineering, The University of Toledo, 2801 West Bancroft Street, Toledo, Ohio 43606, United States; § Siena College, 515 Loudon Road, Loudonville, New York 12211, United States

**Keywords:** Middle School Science, High School Science, General Public, Public Understanding/Outreach, Safety/Hazards, Hands-On Learning, Green Chemistry, Laboratory Management

## Abstract

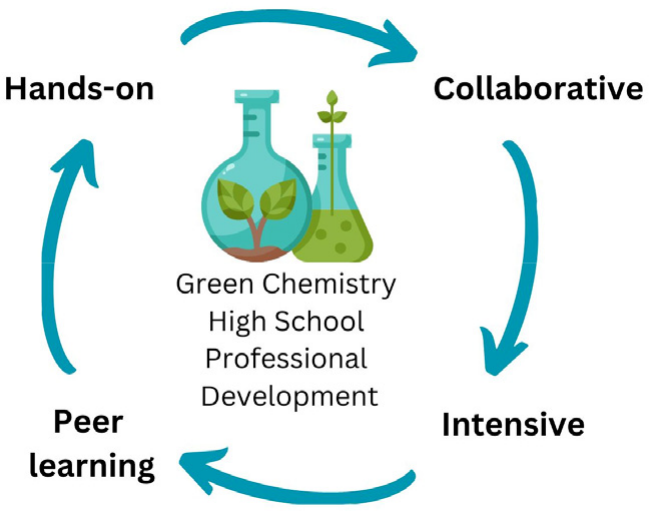

Teaching green chemistry within the K–12 classroom has a positive impact on attitudes and perceptions of chemistry in society for future scientists and professionals, resulting in safer, less hazardous chemistry experiments and demonstrations. The state of New York has taken advantage of the benefits that green chemistry provide in the classroom and is a leader in professional development for high school teachers throughout the state. Between 2011 and 2016, Beyond Benign and Siena College implemented 14 workshops across the state as part of New York’s Department of Environmental Conservation goal of reducing hazardous chemicals in schools. At these workshops, 224 teachers were introduced to green chemistry principles and practices and provided resources for replacing traditional laboratory experiments with alternatives that used safer materials. Two professional development models were implemented, a one-day introductory workshop and a three-day train-the-trainer style in-depth workshop, using collaborative, hands-on, intensive, and peer-learning techniques. In response to a 2021 follow-up survey, participants shared that they continue to use skills from the professional development they received and reported sharing about green chemistry with peers, parents, and administrators. The long-term engagement of the participants indicates that successful models were implemented to provide a path to develop teacher leaders. Professional development models are presented herein for sharing best practices and approaches for training high school teachers on green chemistry, providing numerous benefits to both teachers and students in high school classrooms.

## Introduction

There is a shift within industrial chemistry to design and produce chemical products and processes in a manner that promotes human and environmental health.^1^ The industrial market for greener chemicals is projected to reach 217.18 billion USD by 2029.^2^ Despite this growth, there remains a gap within our educational systems to effectively prepare students to design greener, safer chemicals.^3^ To support the growing demand for sustainable chemicals, green chemistry should continue to be integrated throughout the chemical education pipeline. There is a growing body of resources for green chemistry at the undergraduate level, particularly in the organic chemistry courses.^4−8^ In addition, numerous faculty and professionals have created new courses,^9,10^ resources, books, and additional materials^11^ to aid educators in bringing green chemistry to their courses and laboratories. Even new undergraduate programs have emerged.^12,13^ As a result of these efforts and more, green chemistry principles and practices are increasingly being incorporated into the undergraduate curriculum.^9,10^

In K–12 education, the teaching and practice of green chemistry has grown through informal networks and professional development by organizations^14^ and academic institutions.^15−17^ Beyond Benign, a nonprofit dedicated to green chemistry education, provides open-access curriculum and professional development within its K–12 programs for the elementary, middle, and high school levels, most of which are aligned to Next Generation Science Standards (NGSS) standards.^18^ The ACS Association for the Advancement of Chemistry Teachers (AACT) offers green chemistry curriculum resources,^19^ and academic institutions often connect directly with K–12 teachers to offer innovative green chemistry curriculum and professional development.^15−17^ As a relatively new focus area within chemistry and the physical sciences, curriculum resources and professional development aligned to state and national education frameworks are crucial. Fortunately, green chemistry provides many connections to educational frameworks, providing examples of Crosscutting Concepts, Science and Engineering Practices, and Disciplinary Core Ideas. Green chemistry content provides structure for the framework’s two core ideas: the interdependence of science, engineering, and technology and the influence of STEM on society and the natural world by showcasing the relevance of chemistry to students’ lives and demonstrating the positive and negative impacts of chemistry on the natural world.^20,21^

Teachers need quality professional development for teaching new concepts. The Every Student Succeeds Act (ESSA) provides a federal definition of high quality professional learning with six specific criteria: sustained, intensive, collaborative, job-embedded, data-driven, and classroom-focused.^22^ A report from Frontline Research & Learning Institute revealed that over 80% of the professional development offered by teaching professionals failed to meet the federal definition for four of the six aforementioned criteria.^23^ Additionally, a high percentage of high school chemistry teachers do not have chemistry degrees.^24^ These teachers need more support than those who teach within their field. Successful professional development can impact not only participants but also their students and colleagues.^25^ Professional development for K–12 STEM teachers is an essential support toward the incorporation of green chemistry theory and practice.

### Green Chemistry and Safety in the K–12 Classroom

Green chemistry training can increase safety in the classroom through education about and reduction of hazards. Secondary chemistry educators report feeling that their safety equipment and training is inadequate.^24,26^ While newly hired teachers are assumed to have had proper training in safety during their undergraduate education, this is often not the case, resulting in a group of educators that are ill equipped to recognize hazards.^27−29^ At the present, only seven states include safety training as a part of teacher certification. In addition, some schools do not require certification to teach.^30^ Seventeen percent of school accidents every year are directly related to science instruction and the majority of laboratory accidents result from failing to recognize hazards.^31,32^ One experiment alone, the rainbow flame test, resulted in a staggering 164 reported injuries between 1998 and 2017 and has resulted in safety alerts from both the ACS and the National Science Teacher Association.^30,33,34^

Though green chemistry principles and practices cannot replace safety training among educators, it can increase safety in the classroom by minimizing hazard.^35^ It has been shown in both industry and academic laboratories that green chemistry reduces risk by reducing inherent hazards presented by the materials at hand.^4−6,36,37^ A common strategy in improving lab safety is to identify hazardous chemicals and replace them, when possible, with less harmful alternatives without altering the curriculum.^38,39^ This allows educators to teach the same content with less risk to themselves and their students. For example, the infamous rainbow flame experiment can be performed using colored-flame birthday candles rather than metal salts and flammable solvents.^40^ Using less hazardous materials, also has the added benefit of reducing hazardous waste streams, which can be costly to remove from schools or end up accumulating in storage rooms.^40,41^ Many household chemicals can also be used in green chemistry experiments, which are typically safer and less expensive than those from a chemical supplier.^30,15^ In this way, green chemistry activities are an economical choice for those educators whose curriculum decisions are affected by cost.^42^

## Project Background

### Green Chemistry Professional Development: Filling a Need

With the growing interest in green chemistry in high schools, Beyond Benign and Siena College ran a series of workshops for high school science teachers to introduce green chemistry concepts and their relationship to the state curriculum. The workshops reported herein have shown marked success and are offered as a model for others. This work started as a larger project by the New York Department of Environmental Conservation (DEC) aimed at reducing the amount of hazardous chemicals throughout New York state schools.^43^ To accomplish this goal, the DEC focused on increasing chemical information literacy and creating chemical hygiene plans alongside chemistry teachers, while also providing training and resources for educators to integrate green chemistry into their curriculum. While similar projects were undertaken by other states in prior years, none had the same level of focus on minimizing hazards through green chemistry laboratories.^31,37^ Since the completion of these workshops, the authors have worked closely with other education professionals to spread this model to develop training opportunities in other regions, such as those at the University of Minnesota.^15^

The New York State DEC was awarded funds as part of the EPA’s Pollution Prevention Grant Program.^44^ As a part of the initial EPA grant, the DEC partnered with four schools between 2011 and 2013 to perform school chemical clean-outs, create chemical hygiene plans, and develop case studies considering the impact of teaching green chemistry in the high school and its impact on hazardous chemical reduction. These published case studies are available from the DEC and will not be the focus of this paper.^43^ Having participated in a Beyond Benign green chemistry workshop in 2009, the NYS-DEC invited them to be their lead educational partner in the Pollution Prevention grant and to facilitate green chemistry professional development throughout the state. Professional development workshops were offered near each case study school and participation was free to any New York teacher. At each workshop, a high school green chemistry guide was provided to participating teachers with quality lab exercises that could replace more hazardous existing curriculum while teaching the same concepts. These guides emphasized drop-in replacement lab activities aligned with the New York state learning standards so that participants could easily see where each fit into their curriculum.

**Figure 1:**
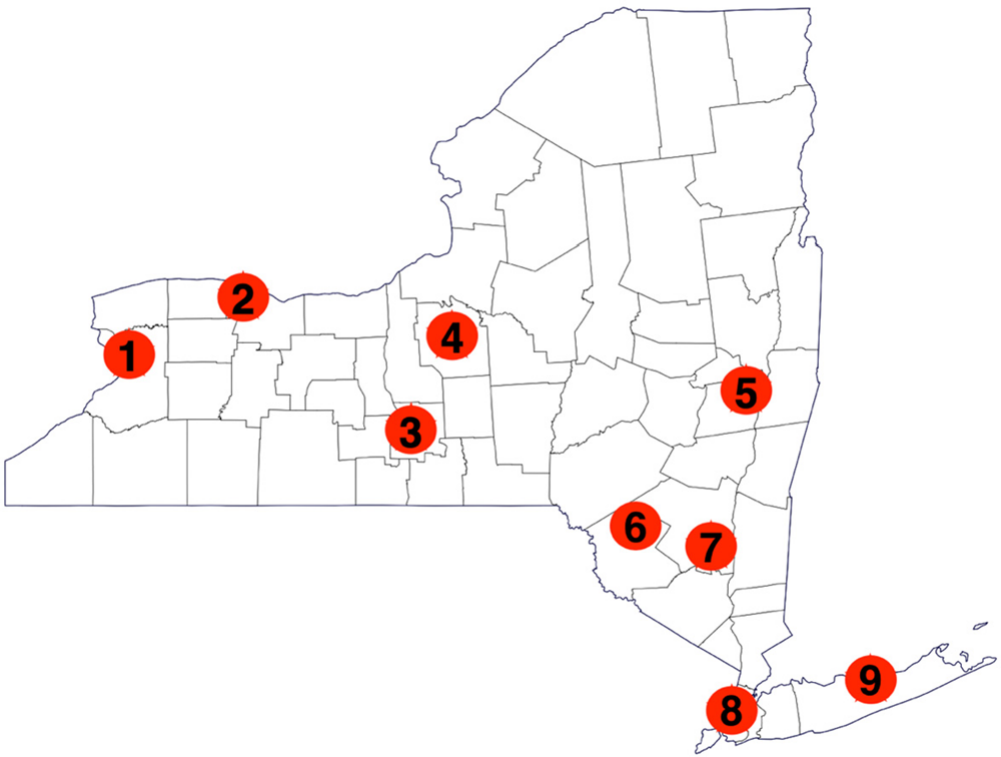
Workshop locations: (1) University at Buffalo, (2) SUNY Brockport, (3) Ithaca College, (4) Syracuse University, (5) Siena College, (6) Liberty High School, (7) SUNY New Paltz, (8) The Cooper Union, and (9) SUNY Stonybrook. Some locations hosted multiple trainings.^43,45^

This project was later expanded through the same EPA Pollution Prevention grant program, as well as an EPA Environmental Benefit Project grant, to include six additional one-day workshops and four three-day workshops from 2012 to 2016 each hosted at a high school, college, or university in New York. All but one of the professional developments were held at a college or university chemistry department. Bringing teachers to college campuses also served as a way of showcasing how green chemistry principles and practices can be carried out throughout the chemical education pipeline. After hosting a Beyond Benign led one-day workshop in January 2012, Siena College began hosting three-day professional development trainings the same summer to meet the demand from teachers for a longer, more in-depth experience. The locations of each workshop can be seen in Figure 1. Beyond Benign led one-day and three-day workshops at each of the locations, with exception of Siena College, where the three-day summer trainings were held and run by Siena College faculty.

### Workshop Preparation

The DEC took the lead in participant recruiting, starting approximately three months in advance, through their existing relationships with school districts throughout the state. All participants received professional development hours, which were granted by either the New York Board of Cooperative Educational Services (BOCES) or Siena College. In addition, teachers at the Beyond Benign led train-the-trainer 3-day workshop were given a stipend for their participation. The cost of the three-day sessions was approximately $1000 to provide rooms, food, and equipment. Stipends for participants and facilitators increase this expense.

Workshop facilitation was led by Beyond Benign or Siena College and included other college faculty, and/or high school teachers using green chemistry in their own classrooms. The workshops typically included two staff and two high school teachers to facilitate the workshops. Workshop sizes were often limited to the room capacity and capped at 25–30 participants to allow for small group activities and enhanced discussion. Laboratory exercises were chosen based on the experience of the specific facilitators at each professional development opportunity, while maintaining the majority of the agenda between workshops.

### Workshop Format

Beyond Benign and Siena College used approaches that included criteria identified by the ESSA, as previously mentioned.^22^ The key criteria for both the one- and three-day professional development workshops included intensive, collaborative, job-embedded, data-driven, and classroom-focused content and activities. Workshops were designed and facilitated alongside classroom teachers to be sure the content was relevant and applicable to a classroom and high school lab setting. The content was often intensive, involving the introduction of several hands-on activities to allow teachers to engage with the materials directly and envision how to bring the activities to their own classroom. Although, both organizations discovered that hands-on instruction was the most beneficial approach for teachers, brief lectures provided useful background in green chemistry principles, hazard assessment, application of state learning standards, and what makes an activity more sustainable. Longer workshops allowed for greater collaboration as teachers often worked together to improve their own teaching laboratory exercises and create safer, less hazardous alternatives. Greener laboratory exercises were often derived from peer-reviewed research or educational articles and modified for the high school classroom setting.

### Workshop Content: One-Day Workshops

Most workshops were single day workshops with the aim of introducing teachers to green chemistry experiments they could use immediately in their classrooms. A sample itinerary of a one-day workshop is provided in Table 1. The introductory session included brief presentations on green chemistry, important definitions, the 12 Principles of Green Chemistry, and the role of green chemistry in the high school classroom. The introductions included an interactive activity to get participants thinking about green chemistry using hands-on approaches and small group discussion. One often used activity involves a set of paper “test tubes” with the symbols of different elements printed on them. Teachers are asked to work in pairs to group the elements in whichever way they see fit (see the Supporting Information for activity). Elements could be grouped in a variety of different ways, from where they fall on the periodic table, their properties, and even alphabetically. What follows is a discussion about how elements and compounds are often grouped by their physical properties, but that toxicity and hazards are rarely considered as properties. This then opens discussion about the need to design chemistry for inherent human and environmental safety.

**Table 1:** Sample Agenda for One-Day Green Chemistry Workshop

8:00–8:30 am	Registration and Breakfast
8:30–9:00 a.m.	Introductory Presentations
9:00–10:15 a.m.	Introduction to Green Chemistry Lecture & Activity
10:15–10:30 a.m.	Move to Laboratory Exercise Stations (4)
10:30–12:30 p.m.	Laboratory Exercise Rotations (30 min per station)
12:30–1:30 p.m.	Lunch
1:30–2:00 p.m.	Resources for High School Teachers
2:00–3:30 p.m.	Laboratory Exercise Rotations (4)
3:30–4:00 p.m.	Wrap-Up: Move to Classroom
4:00–4:30 p.m.	Closing Remarks and Workshop Evaluations

Following the introductory session, the group was given workbooks and safety glasses as they were transitioned to the first round of hands-on laboratory experiments. A sample list of experiments covered throughout the day can be found in Table 2. The participants were split into four groups of 3–5 participants, each with a facilitator, who rotated through 30 min stations. A facilitator at each station presented a brief overview of the experiment and gave the participants time to do the experiment themselves. The groups also spent time networking and brainstorming ways to use the activities in their own classroom.

**Table 2:** Example List of Experiments for Workshops

laboratory experiment^37^	green chemistry and/or safer, less hazardous context
blackberry solar cells	innovation focused experiment featuring dye-sensitized solar cells
Le Chatelier’s principle	replaces traditional cobalt chloride with an iodine-starch complex, using tea
flame test	uses colored birthday candles in place of dissolved metal salts and avoids the use of flammable solvents
reactions lab	hands-on reactions distinguishing single and double displacement, and composition and decomposition reactions
recycling polylactic acid	a hydrolysis experiment transitioning polylactic acid plastic cups to a lactic acid cleaning solution
Taml	catalysis-focused experiment using Fe-Taml, an iron catalyst supported by a tetra-amido macrocyclic ligand (Taml)^46^
exothermic and endothermic reactions	hands-on reactions using household items (liver, hydrogen peroxide, and citric acid (pixy stix)) for thermodynamic reactions
sublimation	sublimation of caffeine from pharmacy tablets; replaces naphthalene (a carcinogen)
catalysts and oxygen	demonstrates the effect of a catalyst on a reaction. replaces manganese dioxide
limonene extraction	experiment extracting limonene from orange peels using liquid carbon dioxide; adapted from college experiment^7^

The afternoon sessions included brief presentations to show teachers how to access the online curriculum,^14^ where they can find other resources that support green chemistry education and increase classroom safety, such as the CleanSweep program^47^ by the DEC. The afternoons also included short presentations by teacher facilitators who spoke to their own experience implementing green chemistry. The participants then headed back into the lab for another round of hands-on experiments through the same format. The workshop concluded with a short debrief, followed by an evaluation.

### Workshop Format: Three-Day Workshops

Beyond Benign and Siena College used slightly different formats for their three-day workshops, but both utilized hands-on approaches and best practices in professional development, building upon the content provided in the one-day workshops. Both organizations found that the longer time provided teachers with a deeper understanding of green chemistry and increased their confidence and ability to teach and implement green chemistry in their classroom. Additionally, more interaction between the teachers led to longer lasting exchanges within the network in the months and years after training. An example of the agenda for a three-day workshop is provided in Table 3. As in the one-day workshops, the focus was on maximizing the number of hands-on experiments.

**Table 3:** Sample Agenda for Three-Day Introduction to Green Chemistry Workshop

Day One	
Morning Session	Registration
	Introductions and Networking
	Introduction to Green Chemistry Principles Lecture & Interactive Activity
	Making Laboratory Exercises Greener Lecture and Activity
Break	Lunch and Informal Discussions
Afternoon Session	Laboratory Session 1:
	Green Information Literacy and Group Work Planning Session
Adjourn	Evening Activities/Dinner

Day Two	
Morning Session	Laboratory Session 2
	Green Chemistry and the New York State Curriculum Lecture
Break for Lunch	
Afternoon Session	Panel Discussion with Past Participants
	Laboratory Session 3
	Group working sessions to green existing exercises or create new green exercises
Adjourn	Evening Activities/Dinner

Day Three	
Morning Session	Group working sessions to green or create laboratory and lecture exercises
	Laboratory Session 4
Break for Lunch	
Afternoon Session	Group presentations: Participants share the outcomes from group working sessions
	Participant Surveys
	Wrap-up and Debrief
Adjourn	

Overall, the format of the multiday workshop was similar to that of the one-day workshop. When not in the lab, presentations provided background and context. This included an introduction to green chemistry principles and practices, how laboratory exercises can be made greener, green information literacy, the alignment of green chemistry to New York state learning standards and a panel discussion featuring educators who have been teaching green chemistry content in their own classrooms. Teachers shared examples of laboratory exercises from their current curriculum that they were to modify, brainstormed ideas with the facilitators and their peers, and worked together in groups toward modifications. The workshop concluded with the participants presenting their work to the group and sharing personal goals for implementing green chemistry followed by with a survey and debrief.

The goal of the multiday workshops was to create networks of teachers to act as ambassadors for green chemistry education by giving them the tools to develop their own green experiments aligned to state learning standards. Years later this network is still influential in the spread of green chemistry education throughout the state of New York.

## Discussion: Workshop Impact

### Participants

**Figure 2:**
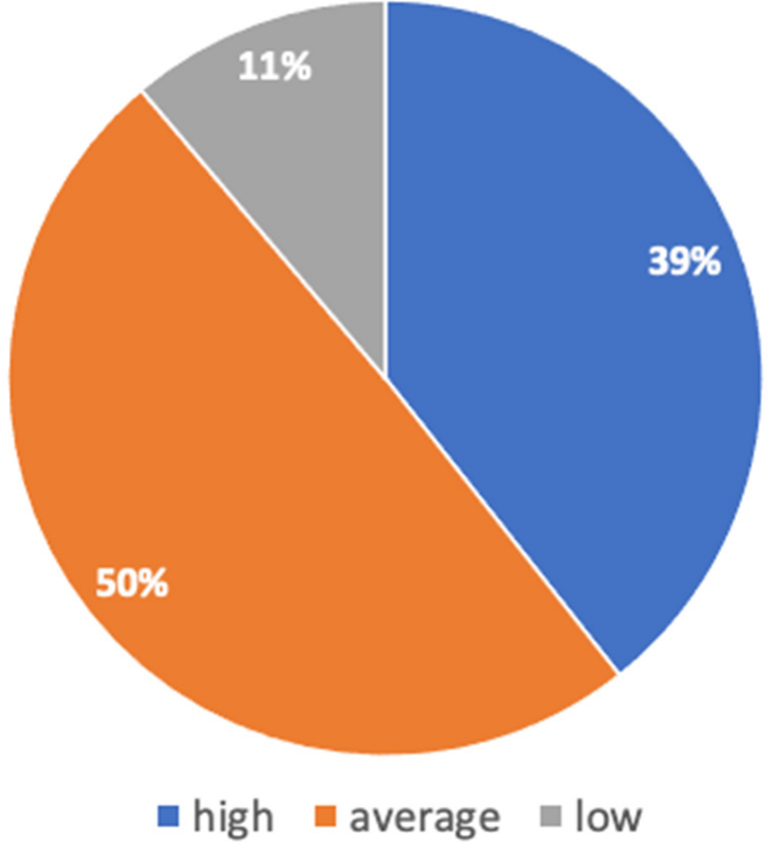
Breakdown of teachers’ employing schools based on high, average, or low need definitions.

A total of 224 teachers from 187 different high schools throughout New York participated in the 14 professional development trainings from 2011 to 2016. Thirty-nine percent of participants were employed at high need schools. High need districts are defined by the National Science Foundations as having a high number of students from low-income families, high teacher turnover, and a high percentage of teachers who are teaching content in which they were not trained to teach.^50^ The implementation of green chemistry practices may have the greatest impact at high need schools due to the cost savings and reduced hazardous waste generation. The remaining teachers were employed at average need and low need schools (Figure 2).

### One-Day Workshop Postworkshop Teacher Reflections

Teachers were asked to take a postworkshop survey at the conclusion of each training. The specific survey questions differed based on workshop length and host. For the one-day trainings, the compiled results of each postworkshop evaluation were provided in the DEC case studies but the overall themes will be summarized here.^43^ In all cases, the most appreciated aspects of the workshops were the time spent working hands-on in the lab trying new experiments. Additionally, the majority of teachers liked the practicality and simplicity of the drop-in replacement laboratories and planned to implement experiments into their own classrooms. Participants also reflected that networking with their peers was a major highlight of the trainings.

Trends also appeared when reviewing constructive feedback that was incorporated in the design of the longer workshops. In each case, teachers shared that having access to the workshop materials prior to the training would allow them to come better prepared. There was also a common reflection that teachers would like more depth on topics, including the environmental and toxicological hazards of common chemicals, as well as more information on chemical hygiene and management. Participants also suggested laboratory exercises covering additional topics not incorporated into the training. Some teachers desired more time spent discussing how to modify existing laboratories and suggested a list of greener chemical substitutions be made available.

### Three-Day Workshop Postworkshop Teacher Reflections

The three-day workshop was designed with the teachers’ suggestions in mind. Some of the topics suggested by the teachers were added to the three-day workshop. Feedback relating to chemical safety and inventory management, beyond the context of specific laboratories, show the demand for the work the DEC was doing. Despite the wish from participants for a green chemical substitutions list, the authors feel that a better approach is to train the teachers to consider each reaction or lab exercise individually, as there are rarely two chemicals that are universally interchangeable.

Similar reflections from the one-day workshops were observed in the postworkshop surveys regarding the three-day training. The most helpful parts of these workshops were reported to be the collaborative discussions with peers and the hands-on time in the lab. Over 90 percent of participants reported that they felt prepared to implement green chemistry into their classrooms and laboratories. As in the one-day workshops, teachers expressed interest in adding more laboratory exercises to cover additional topics and wished for more information on proper chemical hygiene and disposal. Participants unanimously reported that they would recommend the workshop to a colleague.

Nine participants (out of 11) who attended a three-day workshop at SUNY New Paltz were surveyed in 2017, one year after the training, to assess the ways in which they were incorporating green chemistry into their teaching. All respondents reported practicing green chemistry at least once per month, with most incorporating some green chemistry content in their classrooms at least once per week. All participants agreed that their students could identify how green chemistry relates to the real world, and 89% agreed that their students could identify the principles of green chemistry. Of the experiments presented in their workshop, Le Chatelier’s Principle,^19^ solubility,^48^ reactions,^49^ and freezing point determination^51^ were the most used by the teachers following the training. Most of the teachers also reported reducing or eliminating compounds of concern as a result of the training. Encouragingly, 100% of respondents stated that they planned to further increase their knowledge of green chemistry.

### Long-Term Postsurvey Teacher Reflections

Beyond Benign and Siena College invited teachers from the 2011–2016 trainings to complete a follow-up survey in 2021 on their incorporation of green chemistry into their work since their participation. All 224 participants were contacted; 41 current teachers and 1 retired teacher completed the survey. Overall, these 42 participants reach over 3000 sary students per year, providing a safer learning environment and incorporating the concepts presented in these conferences. The participants found the experience very valuable with one participant claiming that “the training really changed my philosophy about the lab experience as I was starting my career as a teacher. As a corporate scientist, I gave little thought to choices in materials of methods and compensated with budgeting for whatever was needed in terms of waste disposal, power consumption, or whatever.”

**Figure 3:**
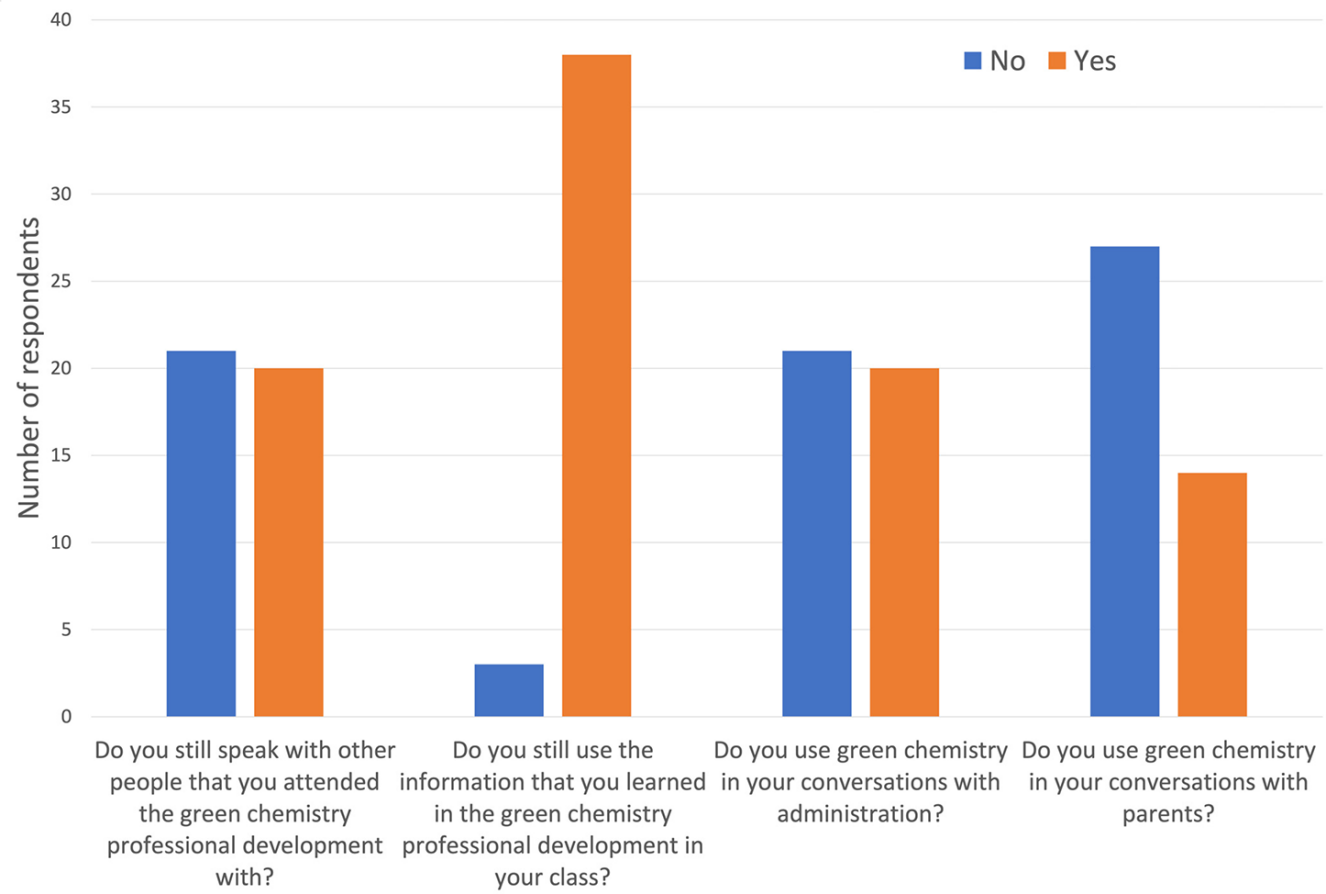
Summary of key questions from 2021 survey data from 42 past participants of the New York state high school workshops between 2011 and 2016.

Figure 3 includes responses to key questions from the survey. Over 90% of respondents indicated they use what they learned in the workshops in their classrooms and about half of the respondents remain connected with colleagues from the workshop. It was also found that green chemistry allows teachers the opportunity to shine a positive light on chemistry, therefore reducing “chemophobia” of school administrators, parents, and community members. Almost 50% have discussed green chemistry with their administrators and find that it helps in budgeting discussions. One third of respondents even use green chemistry in their discussion with their students’ parents. Often, educators mention this on parents’ night to highlight their dedication to the safety of their students while still teaching the traditionally important scientific principles. One participant runs a low hazard experiment with the parents to demonstrate the ability for students to learn with little to no risk.

Many participants are actively running green experiments from the workshop and discussing the reduced hazards with students. One participant elaborated on this concept, saying, “I am consistently evaluating the materials and methodology that I use for the laboratory experience with my students. I try to work strictly with benign materials. . . and I encourage my colleagues to do the same. In addition, I have students do a regular HSE [health, safety, and environment] evaluation for each lab. They discuss health hazards, safety hazards and impact on the environment.” The high implementation rate, is possible due to adaptable experiments that teachers can take back to their own classrooms.

When asked what was most useful from the workshop, 67% of respondents stated the hands-on laboratory activities, 21% indicated lectures, and 12% said that the networking was the most useful part of the experience. One participant highlighted this saying: “The contacts that I made. . . are still mostly part of my regular communication outside the school district. I appreciate sharing materials with other like-minded colleagues outside my school and hearing new things that they are doing as well.”

In addition, the workshops provided time for the teachers to think about waste minimization and limiting exposure to hazardous chemicals. Teachers indicated relating knowledge of human health and the environment to their students. One participant acknowledged this saying, “When discussing lab development and safety [in my classroom], we have a conversation about the importance of minimizing risk to both the scientist and the environment whenever possible.”

## Discussion: Keys to Success

### Key Partners

The partnership between Beyond Benign, Siena College, and the DEC was key to the success of these workshops. The DEC’s connections and positive reputation within schools across the state allowed for the high level of engagement with teachers. The DEC used local BOCES as primary contacts and recruitment tools. BOCES is a New York State program focused on shared educational services across districts.^52^ This resource helped to generate perceived value, so that secondary educators were able to consider participation. Additionally, facilitators joining list-serves, being active and recognizable in the community, and speaking at local, regional, and national conferences about green chemistry were leveraged to establish contacts in the community. Upon completion of the program, past participants became effective recruiters by offering personal insight, sharing resources, and confirming the program’s success and benefits. Utilizing the requirement for educators to complete professional development also provided critical traction toward generating participation in trainings. As an example, Siena College offered training for the local catholic schools at an annual diocese day of training.

### Hands-On Learning

Siena College and Beyond Benign, working in parallel, discovered the most productive use of time were hands-on instruction in green chemistry experiments that teachers could implement in their schools. However, lectures were necessary to provide background in green chemistry principles, hazard assessment, application of the state learning standards, and discussion on what makes an activity more sustainable. Further, inviting current teachers who were actively employing green chemistry in their classroom to share their experiences was helpful and well-received, as teachers often prefer to hear from those actively in the classroom.

A highly effective session within the workshop was the time dedicated toward the development of novel laboratory exercises or greening existing laboratory experiments. The active discussion among the teachers not only improved targeted experiments within the curricula but also generated a network of colleagues to provided additional peer support. The workshop organizers supported the laboratory investigation by providing chemicals, supplies, equipment, and expertise. It was especially helpful to have an organizer with a background in toxicology or chemical hygiene to advise. In spending time brainstorming how to modify their own laboratory exercises, teachers shared the decision-making process resulting in hazardous waste source reduction and energy conservation. Then, the teachers discussed the decision-making process with their own students when the experiment was conducted in their classrooms.

### Summary and Lasting Impact

In the years since these workshops throughout New York, green chemistry education has continued to grow in high schools across the state. As a single teacher can reach over one hundred students annually, the impact of a single training can be quite large over time. The 42 respondents to the most recent survey continued to implement green chemistry in their classroom and exposed over 18,000 students, based on 41 active teachers (one retired teacher responded) teaching an average of 80 students annually, to green chemistry between 2016 and 2022 alone. Since workshops began running in 2011, the number of students who learned green chemistry principles and practices from trained teachers is likely much larger. These numbers also reflect a reduction in student and teacher exposure to hazardous materials.

Some of the original participants have continued to maintain relationships with Beyond Benign and actively seek further training in green chemistry. The train-the-trainer model used in New York helped lay the foundation for Beyond Benign’s Lead Teacher Program, through which current K–12 teachers partner with Beyond Benign in leading professional development and developing curriculum.^53^ Of the seven Lead Teachers from New York state, four are alumni of the 2011–2016 workshops. These educators have actively been involved in connecting their peers to green chemistry resources through running workshops at the annual Science Teachers Association of New York (STANYS) conference and the New York City STEM Institute.^54,55^ The initial Lead Teachers who are also a part of the New York State Master Teacher Program have led green chemistry trainings for other Master Teachers, increasing the number of high impact educators who are equipping the next generation of green chemists and increasing safety within their classrooms. The teacher network that has evolved out of the New York workshops provide support to isolated chemistry teachers and those that are teaching chemistry despite it not being their primary field of training.^24^

New York Lead Teachers designed a guide to align New York standards with green chemistry curriculum.^56^ As these standards are similar to NGSS, this guide can be easily adapted for the 43 other states that have adopted NGSS or have developed state standards similar to NGSS.^57^

Green chemistry workshops for high school teachers have since been implemented elsewhere by the authors and others. Beyond Benign has led green chemistry teacher trainings across the country in partnership with Lead Teachers and additional academic partners. Other universities have hosted similar workshops for teachers, including the University of Minnesota, facilitated in part by Beyond Benign Lead Teachers from Minnesota.^15^ The workshops included similar green chemistry replacement laboratories and emphasized hands-on learning. Universities are particularly well-suited to host future workshops within their chemistry departments, as they may donate laboratory space and stockroom personnel time and recruit within their existing connections to teachers from their region.

Green chemistry and sustainability must be integrated throughout the education continuum. The next generation of scientists must be trained with the knowledge and skills to serve society in their professional capacity through the articulation, evaluation, and employment of methods and chemicals that are benign for human health and the environment. Early education is central to creating lasting change in chemistry education to support green technological innovations that result in less hazardous materials, products and processes for a sustainable, healthy society.

## Supporting Information Available

Group the Elements activity (PDF, DOCX)
