# Supplementary material for: Green Chemistry Teacher Professional Development in New York State High Schools: A Model for Advancing Green Chemistry
Source: J Chem Educ. 2023 May 3;100(6):2224–32. doi: 10.1021/acs.jchemed.2c01173 (PMC10269322; doi:10.1021/acs.jchemed.2c01173)

Supporting Information

Green Chemistry Teacher Professional Development in New York State High Schools: A Model for Advancing Green Chemistry

Amy S. Cannon,[1] Kate R. Anderson,[1] Mollie C. Enright,[2] Donia G. Kleinsasser,[3] Ann R. Klotz,[3] Natalie J. O'Neil,[1] Lucas J. Tucker*[3]

[1]Beyond Benign, 18 Church Street PO Box 1016, Wilmington, MA, USA 01887, [2]Department of Chemistry and Biochemistry, School of Green Chemistry and Engineering, The University of Toledo, 2801 W. Bancroft St., Toledo, OH, USA 43606, [3]Siena College, 515 Loudon Rd., Loudonville, NY, USA 12211; ltucker@siena.edu

Included in the supporting information is the "Group the Elements" activity mentioned within the manuscript. The activity is a simple way for introducing concepts of green chemistry and prompting discussion.

***Group the Elements* – Facilitator Guide**

Overview: Invite participants to begin thinking about the need for green chemistry by grouping a set of elements by their properties. Participants may group the elements by whatever properties they wish. At the end, the group discusses how toxicity, natural abundance, and environmental impact are inherent properties of materials that are often not discussed when doing chemistry.

Goal: Introduce concept of direct vs indirect hazards of chemistry.

Materials: Printed test tubes (1 set per pair or group) (See Supporting Figure 1).

Facilitator prep: Cut paper test tubes.

Additional resources:

- Periodic Table of Endangered Elements by the American Chemical Society Green Chemistry Institute: https://www.acs.org/content/acs/en/greenchemistry/research-innovation/endangered-elements.html

Activity:

- Tell the participants that you will be handing out stacks of test tubes and each test tube represents a different element. Instruct the participants to group the elements together in whichever way they wish. There is no right or wrong way to group them.
- Break out the participants into smaller groups or into pairs and pass out the test tubes.
- Allow a few minutes for them to do the activity. Once it seems like everyone is done, call the participant's attention back together.
- Invite participants to share how they grouped their elements. You may wish to write their criteria on a white board or flip chart, if available.
    - Common groupings may include:
        - Alphabetical
        - One letter vs Two letter symbols
        - Metals
        - Non-metals
        - Noble gases
        - Atomic masses
        - Melting point
        - Conductivity
        - Flammability
- If toxicity, environmental impact, or natural abundance of elements have not been mention, reflect on the list of criteria the class has presented and point out the inherent properties that have been listed.
- Ask the group if anyone had considered other properties inherent to each element, like toxicity, natural abundance, or environmental impact.

  - If we group by toxicity - mercury, lead, and cadmium are all toxic heavy metals, while many of the other elements have low toxicity, like oxygen, iron, and the noble gases.
  - If we group by natural abundance – oxygen, silicon, aluminum, and iron are the four most abundant elements on earth; helium and silver are very low availability with serious threats to their supplies within the next 100 years.
  - If we group by environmental impact (related to both toxicity and natural abundance) – mercury and cadmium are toxic to the environment, mining of elements in low supply often involves a huge carbon footprint and intensive refining processes.
- In closing, reflect that as chemists or chemistry teachers, we're not typically trained to think of environmental impact and toxicity as being essential to the choices we make in the lab, but training ourselves to consider these aspects of our chemistry can have big impacts both in and out of the lab.

Supporting Figure 1. Element test tubes

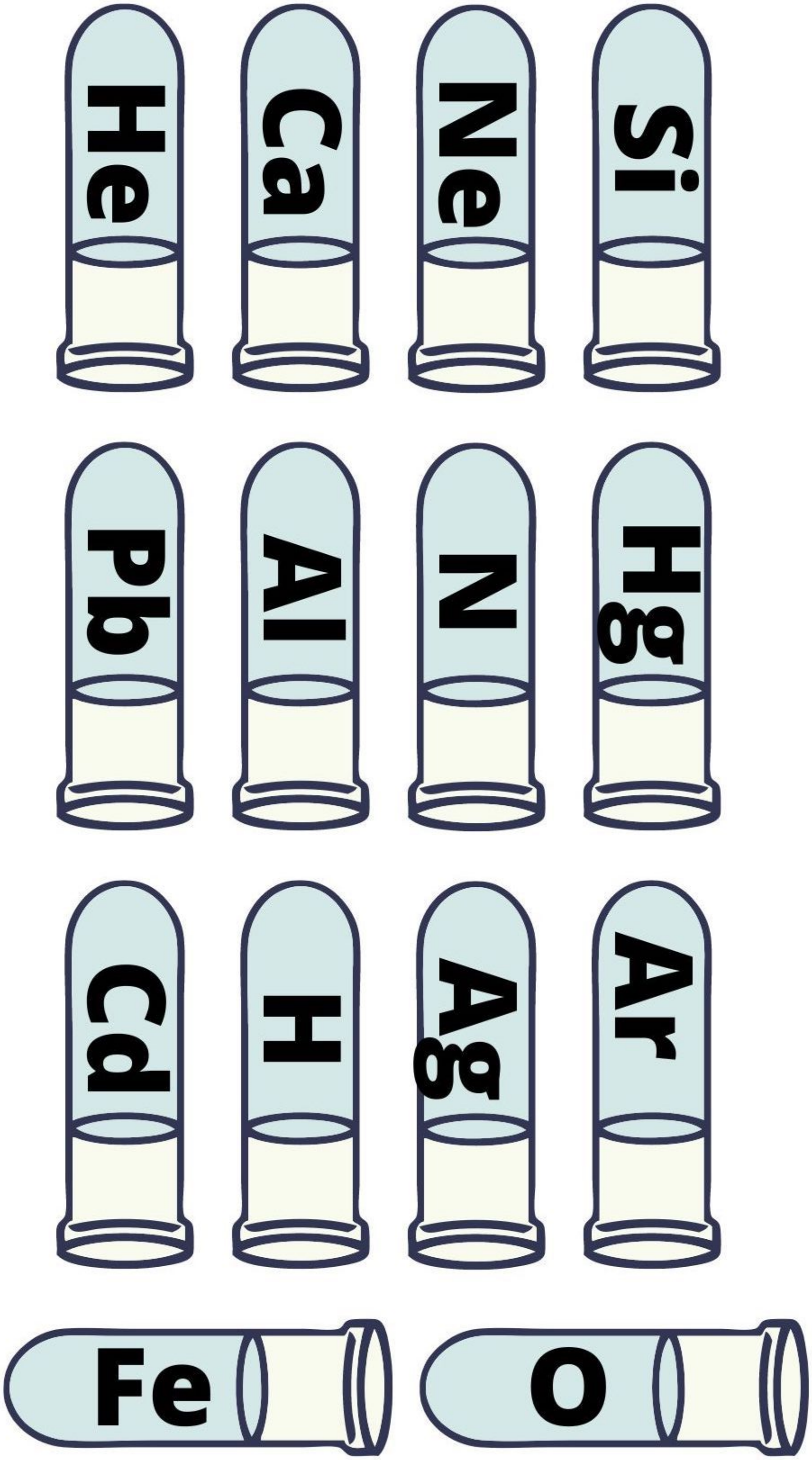

Supplement: Supplementary file 1 — ed2c01173_si_001.pdf [file ed2c01173_si_001.pdf]
